# Successful pregnancy in each half uterus cavity of the septate uterus after transferring three embryos in one half-cavity: a case report

**DOI:** 10.1186/1757-2215-6-56

**Published:** 2013-08-07

**Authors:** Xiaoqiao Guo, Xiaofang Sun, Haiyan Xu, Shasha Si, Bolan Yu, Jianqiao Liu

**Affiliations:** 1 Reproductive Medicine Center, the Third Affiliated Hospital of Guangzhou Medical University, Guangzhou Medical University, Guangzhou, Guangdong Province, China; 2 Key laboratory for Major Obstetric Diseases of Guangdong Province, the Third Affiliated Hospital of Guangzhou Medical University, Guangzhou Medical University, Guangzhou, Guangdong Province, China

**Keywords:** Müllerian duct anomalies, Septate uterus, In vitro fertilization-embryo transfer(IVF-ET)

## Abstract

The incidence of Müllerian duct anomalies in women is rare, associating with urinary tract anomalies such as horseshoe or pelvic kidney, renal agenesis, duplication of the collecting system, or ectopic uterus. Septate uterus is one type of congenital uterine anomalies, in which there is a septum from the fundus to the cervix. Currently, it is believed that hysteroscopic metroplasty is a leading choice for patients if their recurrent spontaneous abortion is resulted from septate uterus. But, some people refuse to have a surgery, and successfully delivery. In this case report, we report a successful pregnancy with two gestational sacs, one in each half-cavity, in a woman with septate uterus after transfer of three embryos into one half–cavity. Finally, the women gave birth to two healthy babies in our hospital.

## Background

Septate uterus is one kind of uterine malformations, in which there is a septum from the fundus to the cervix. Complete septum is that the uterine cavity and endocervical canalis is completely separated into two components, no matter equal or unequal, while partially separated one is called the incomplete septum [1].

Female internal genital origins from Müllerian duct, of which the middle part and tail develops into the uterus. A uterine septum is a result of abnormal lateral fusion of the paired Müllerian ducts during early embryologic development. Müllerian anomalies may also associate with urinary tract anomalies such as horseshoe or pelvic kidney, renal agenesis, duplication of the collecting system, or ectopic uterus [2].

The incidence of Müllerian duct anomalies in women is about 1%–3% [3]. Septate uterus is one of the most common types of congenital uterine anomalies [3]. It has been reported that septate uterus is associated with infertility, spontaneous abortion, premature delivery, and fetal abnormalities [4,5]. For instance, *Heinonenonce* reported that three infants from women with septate uteri had limb anomalies [6]. However, some patients with septate uterus still can delivery normal infants after in vitro fertilization-embryo transfer(IVF-ET). In this case report, we reported a successful pregnancy with two gestational sacs, one in each half-cavity, in a woman with septate uterus after transfer of three embryos into one half–cavity. Finally, the women delivered two healthy babies in our hospital.

## Case presentation

**Figure 1:**
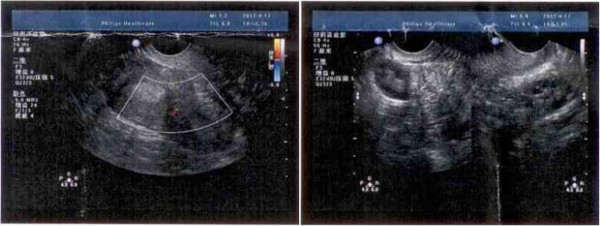
**Longitudinal scan: the transverse diameter of the uterine is significantly wider than normal uterus, two endometrial echoes were showed.** The thickness of the left endometrium is 0.8 cm, the right is 0.9 cm, and there is no abnormal echo (left) and the bilateral annex areas are normal (right).

A 32-year-old nulligravida woman was admitted to our department for the treatment of the primary infertility, which was diagnosed as polycystic ovary syndrome and septate uterus. She refused to have a surgery to do metroplasty when she was informed that the septum might be the main reason for the infertility. She had been married for 3 years and received several ovulation induction cycles with human menopausal gonadotropin (HMG) and Human Chorionic Gonadotropin(hCG) for timed intercourse or IUI but without success. The ratio of LH/FSH was higher than 2 and the level of androgen was normal at the year of 2007. B-mode Ultrasonic showed anovulation and a complete septate uterus that extended to the cervix and left renal agenesis but bilateral annex was normal (Figure 1). Hysteroscopy revealed that the right half-cavity of the uterus was normal and, the right fallopian tube was opening and no cervix on the left cavity.

In the preceding 2 years, the patient received treatments with Artificial Insemination and IVF-ET in other hospitals, but all failed. She came to our hospital and underwent an IVF cycle when she was 32-year-old. Gonadotropin-releasing hormone (GnRH) agonist was used at the 22th day of her menstrual cycle. Recombination-Follicle-Stimulating Hormone (FSH) was administered for ovarian stimulation starting with 150 IU/day on the third day of her next period. The dose of FSH was adjusted to 75 IU/day after 8 days and HMG was applied at a dose of 75 IU/day. On the 11th day of ovarian stimulation cycle, Intramuscular 10,000 IU hCG (Profasi; Serono) was injected for inducation of the final oocyte maturation. After 36 hours, the patient underwent an oocyte retrieval surgery and 12 oocytes were retrieved. Totally 11oocytes were inseminated, of which 9 fertilized normally. Two days after the oocyte retrieval, 3 cleavage stage embryos were transferred into the right uterus cavity. For luteal support, intramuscular progesterone (P) at a dose of 40 mg/day was started on the day of oocyte retrieval.

**Figure 2:**
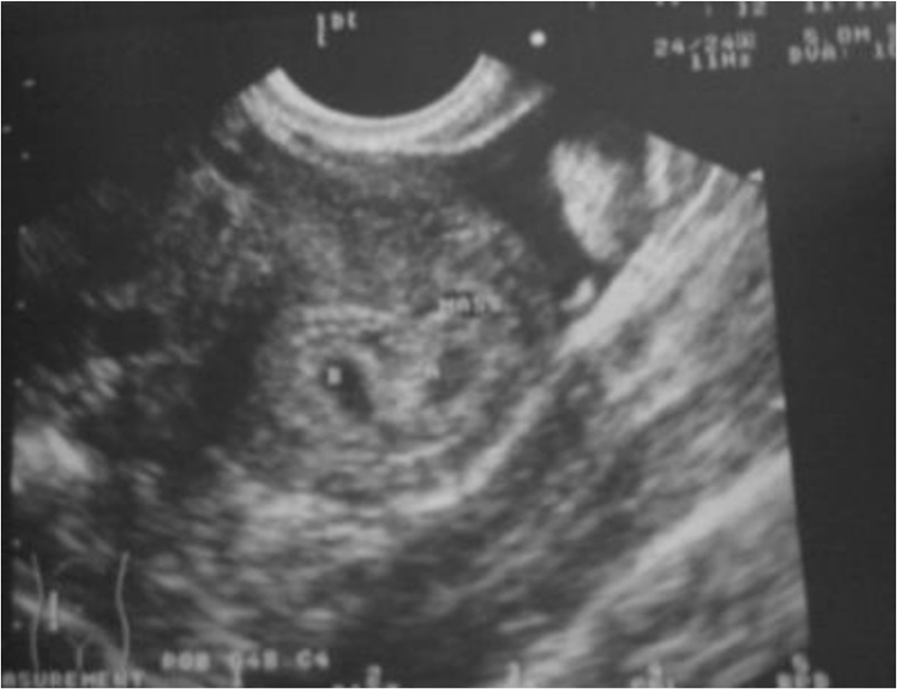
The transvaginal ultrasound revealed two different gestational sacs in uterine cavity and one had the fetal heart beat.

Two weeks later after embryo transfer, the test of urine hCG showed a positive pregnancy. An early transvaginal ultrasound at 5^+^ weeks revealed two gestational sacs in uterine cavity and one had the fetal heart beat (Figure 2). At 7^+^ weeks of gestation, transvaginal ultrasound confirmed both sacs had fetal heart beat. At 34 weeks of gestation, two male infants were delivered without complications by cesarean section.

## Discussion

Uterine malformations may associate with urinary tract malformations, which can be evaluated by transvaginal ultrasound scan. In this case, the patient had a septa and saddle uterus and unilateral renal abnormalities, which can be easily found by ultrasound examination. It has been found that hysteroscopic septum resection of septate uterus is the main reason of recurrent spontaneous abortions and/or infertility [7-11]. *Mollo* et al. [8] reported that hysteroscopic metroplasty improved fecundity in unexplained infertility patients with septate uterus. Their data indicated that the pregnancy rate in women with metroplasty surgery was 38.6%, which was higher than that in women without surgery (20.4%). The live birth rate was also higher in the patients who had surgery (34.1%) than that (18.9%) in the patients who did not have surgery. *Paradisi* et al. [12] reported that the hysteroscopic metroplasty was a simple, safe, and rapid surgical procedure that can improve the pregnancy.

Currently, it is believed that hysteroscopic metroplasty is a leading choice for patients if their recurrent spontaneous abortion is resulted from septate uterus. *Fedele* et al. [13] found that the endometrium of the septa had decreased number of glandular ostia, the ratio between ciliated and non-ciliated cells, supporting that the abnormal endometrium on the lateral wall was a cause of primary infertility.

In this case, we transferred three embryos to one half uterus cavity, but surprisingly, we found that each half uterus cavity had a pregnancy sac. It was not clear how this happened, but a few of possibilities may exist. Firstly, there may be a tiny channel between the two half of uterus cavities, which was not found with hysteroscopy. Secondly, the patient got a natural conception in the other half cavity during the IVF cycle. After reviewing the patient's medical records there were five large follicles (>15 mm in diameter) and three small follicles (<11 mm in diameter) in the left ovary on the hCG day. However, we found only 5 follicles in the left ovary before egg retrieval. Some follicles might ovulate and it was possible that the oocytes were fertilized if there was a sexual intercourse. This may cause natural conception. Third, it is difficult but possible that a long distance migration of an embryo from half uterine cavity through the ipsilateral fallopian tube to the contralateral tubal and then to the uterine cavity. Finally, it may be also possible that one or two embryos drop at the cervix during the ET, and then the embryos migrated to the other half cavity.

## Consent

Written informed consent was obtained from the patient for publication of this Case report and any accompanying images. A copy of the written consent is available for review by the Editor-in-Chief of this journal.

## Abbreviations

IVF-ET: In vitro fertilization-embryo transfer; HMG: Human menopausal gonadotropin; hCG: Human chorionic gonadotropin; LH: Luteinizing hormone; FSH: Follicle stimulating hormone; GnRH: Gonadotropin-releasing hormone; P: Progesterone; IUI: Intrauterine insemination.

## Competing interests

The authors declare that they have no competing interests.

## Authors’ contributions

JL drafted the manuscript. XS and BY supervised the study. XG writed this article and involved in design. HX provided the clinical data. SS involved in interpretation and data preparation. All authors had read and approved the final manuscript.
